# The Effect of Repetitive Transcranial Magnetic Stimulation on Cognition in Diffuse Axonal Injury in a Rat Model

**DOI:** 10.3390/neurolint16040052

**Published:** 2024-06-25

**Authors:** Hyeong-Min Kim, Hyun-Seok Jo, Eun-Jong Kim, Ji-Min Na, Hyeng-Kyu Park, Jae-Young Han, Ki-Hong Kim, Insung Choi, Min-Keun Song

**Affiliations:** Department of Physical & Rehabilitation Medicine, Chonnam National University Medical School & Hospital, Gwangju 61469, Republic of Korea; freshsky986@naver.com (H.-M.K.); jjm923@naver.com (H.-S.J.); trueisone@hanmail.net (E.-J.K.); wlals0928@naver.com (J.-M.N.); phk1118@naver.com (H.-K.P.); white--fish@hanmail.net (J.-Y.H.); manutalk@hanmail.net (K.-H.K.)

**Keywords:** diffuse axonal injury, cognition, repetitive transcranial magnetic stimulation

## Abstract

Diffuse axonal injury (DAI) following sudden acceleration and deceleration can lead to cognitive function decline. Various treatments have been proposed. Repetitive transcranial magnetic stimulation (rTMS), a non-invasive stimulation technique, is a potential treatment for enhancing neuroplasticity in cases of brain injury. The therapeutic efficacy of rTMS on cognitive function remains unconfirmed. This study investigated the effects of rTMS and the underlying molecular biomechanisms using a rat model of DAI. Sprague–Dawley rats (n = 18) were randomly divided into two groups: one receiving rTMS after DAI and the other without brain stimulation. All rats were subjected to sudden acceleration and deceleration using a DAI modeling machine to induce damage. MRI was performed to confirm the DAI lesion. The experimental group received rTMS at a frequency of 1 Hz over the frontal cortex for 10 min daily for five days. To assess spatial memory, we conducted the Morris water maze (MWM) test one day post-brain damage and one day after the five-day intervention. A video tracking system recorded the escape latency. After post-MWM tests, all rats were euthanized, and their brain tissues, particularly from the hippocampus, were collected for immunohistochemistry and western blot analyses. The escape latency showed no difference on the MWM test after DAI, but a significant difference was observed after rTMS between the two groups. Immunohistochemistry and western blot analyses indicated increased expression of BDNF, VEGF, and MAP2 in the hippocampal brain tissue of the DAI-T group. In conclusion, rTMS improved cognitive function in the DAI rat model. The increased expression of BDNF, VEGF, and MAP2 in the DAI-T group supports the potential use of rTMS in treating cognitive impairments associated with DAI.

## 1. Introduction

Traumatic brain injury (TBI) results from various causes, including traffic accidents, falls, and blunt trauma. Approximately 500 per 100,000 individuals in the United States require hospitalization due to TBI [1], with their prognosis significantly influenced by their initial state of consciousness [2]. Cognitive impairments after TBI are common and variable [3]. TBI significantly impacts patients’ activities with cognitive, behavioral, psychosocial, and physical impairment [4]. After TBI, cognitive impairment substantially affects the ability to resume social activities [5]. Notably, the decline in cognitive function following TBI is often linked to diffuse axonal injury (DAI) resulting from abrupt acceleration and deceleration. Various interventions are available to restore cognitive function in patients with DAI. These may encompass cognitive rehabilitation, psychotherapeutic methods, pharmacological treatments, and non-invasive brain stimulation techniques [6,7,8,9], all aiming to enhance neuroplasticity following brain injury.

Non-invasive brain stimulation methods, including repetitive transcranial magnetic stimulation (rTMS), transcranial direct current stimulation, and alternating current stimulation, improve functions in brain injury cases. Applying rTMS at frequencies of 5 Hz [10] and 10 Hz [11] to the injured motor cortex can boost motor function neuroplasticity in stroke patients. Additionally, 1 Hz rTMS has also been shown to aid neurological recovery [10,12]. In TBI rat models, rTMS over the cortex can increase regional blood flow and induce synaptic reorganization, a form of neuroplasticity [13,14,15,16,17]. A study applying various rTMS protocols to normal rats and assessing the effects using functional magnetic resonance imaging reported that 1 Hz low-intensity rTMS bilaterally decreased resting-state activity in the somatosensory cortex, motor cortex, and hippocampus [18]. However, the molecular biomechanisms underlying recovery following DAI treated with rTMS remain unclear. Although cognitive rehabilitation aims to mitigate cognitive decline, it often necessitates an extended duration and may not wholly restore prior functional abilities. Although rTMS is anticipated to enhance cognitive function in patients with DAI, its therapeutic efficacy is yet to be confirmed.

This study aimed to evaluate the effect of rTMS on cognitive function in a DAI rat model.

## 2. Materials and Methods

### Experimental Subjects

All animal protocols adhered to the guidelines set by the Chonnam National University Animal Care and Use Committee. The Chonnam National University’s Institutional Animal Care and Use Committee (CNU IACUC-H-22020) approved the study protocol. We used forty male Sprague–Dawley rats, aged 10 weeks and weighing 300 ± 50 g (Samtako, Osan, Republic of Korea). The rats were housed at 23.0 ± 1.0 °C and 50 ± 5% humidity under a 12 h alternating light–dark cycle. They had unrestricted access to water and food in the Chonnam National University Animal Care Laboratory.

## 3. Methods

### 3.1. DAI Rat Model

**Figure 1:**
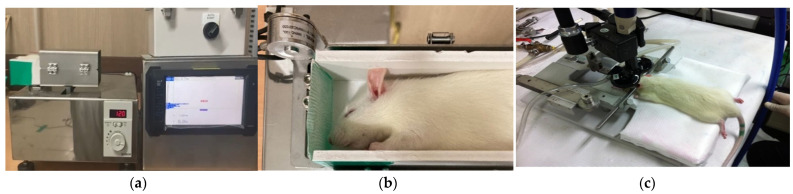
Modeling a diffuse axonal injury (DAI) rat and applying repetitive transcranial magnetic stimulation (rTMS). A DAI rat model was created using an acceleration and deceleration modeling machine (**a**,**b**) with an impulse of 3.8 kg× m/s for 15 s, following anesthesia with isoflurane gas. (**c**) The rats received rTMS treatment using a figure-eight coil (inner diameter: 2.5 cm; outer diameter: 5 cm; Magstim Rapid2, MagStim^®^, Whitland, Dyfed, UK) at an intensity of 50% of the resting motor threshold and a frequency of 1 Hz for 10 s, with 50 s of rest between sets. Treatment consisted of 10 sets daily for five consecutive days.

We divided 18 ten-week-old male Sprague–Dawley rats into two groups: the DAI-T group, which received rTMS after DAI, and the rats receiving no stimulation after DAI (DAI-NT group). Each rat was subjected to sudden acceleration and deceleration, with an impulse of 3.8 kg × m/s for 15 s, to induce brain damage (Figure 1).

### 3.2. Sample Size Calculation

We followed a previous rTMS study for sample size calculation [19]. The sample size was estimated using G*Power 3.1 software. Allowing a type 1 error of 5%, α = 0.05 with the power of 60%, β = 0.4, we calculated a needed sample size of nine rats per group.

### 3.3. Repetitive Transcranial Magnetic Stimulation

Rats in the DAI-T group underwent rTMS treatment (Magstim Rapid2, MagStim^®^, Whitland, UK). The resting motor threshold used to set the TMS intensity was measured from the gastrocnemius muscle. Previous studies have suggested that an intensity of 80% of the resting motor threshold affects motor recovery in a stroke rat model [20]. However, we considered inflammation in acute TBI. Another study has shown that low-intensity rTMS reduces inflammation in acute TBI [21]. Therefore, we fixed the intensity at 50% of the resting motor threshold. The frequency was set at 1 Hz for 10 s, followed by a 50 s rest interval per set. Daily stimulation comprised 10 sets and was conducted for five consecutive days. The rTMS was administered targeting both frontal lobes, with stimulation provided in a vertical direction. The rTMS, using a figure-eight coil (inner diameter 2.5 cm; outer diameter 5 cm), was administered to anesthetized rats starting from one day post-DAI. During the experiment, the rats in the DAI-NT group were under the same circumstances as the DAI-T group without non-invasive brain stimulation for five consecutive days.

### 3.4. Morris Water Maze Test

The memory function and spatial learning assessment was conducted using the Morris water maze (MWM) test, as described by Morris et al. [22]. This test involved a circular pool with a diameter of 184 cm and a height of 60 cm, filled with water maintained at a temperature of 22 ± 2 °C. The pool was conceptually divided into four quadrants, with one designated as the target quadrant. Visual cues were placed around the perimeter of each quadrant. Within the center of the target quadrant, a circular escape platform (10 cm in diameter and 38 cm in height) was submerged 1 cm below the water surface.

Before the induction of DAI, all groups underwent three consecutive days of pre-training. The animals were introduced into the maze at random entry points along the wall, distributed evenly around the maze’s perimeter. Upon locating the platform, rats were allowed to remain there for 10 s before initiating the next trial. Rats unable to locate the platform within 120 s were manually placed on it for 15 s to facilitate recognition of its location. Subsequently, the rats were removed from the pool and returned to their cages for a 5 min interval before the commencement of a second trial.

The MWM test was administered the day following the brain injury to assess spatial memory. The rats attempted to locate the platform within a 300 s timeframe. Escape latency, or the time taken to reach the platform, was accurately recorded using an Ethovision Color-Pro^®^ video tracking system (Nodulus, Wageningen, The Netherlands).

### 3.5. Imaging Study

One day after inducing DAI in rats, a brain MRI was conducted. Three rats were used in each group to confirm the DAI lesion. The MRI data were collected at the Douglas Centre d’Imagerie Cérébrale utilizing a 4.7 Tesla Bruker Biospec 47/40 scanner (Bruker, Billerica, MA, USA), equipped with a 35 mm rat and mouse brain surface coil for transmission and a two-channel surface coil array for signal reception (Bruker). Rats were anesthetized with oxygen and isoflurane (4% isoflurane during induction, followed by 2–3% for maintenance). The level of isoflurane was adjusted to keep the breathing rate within 45–65 breaths/min throughout the procedure, and warm air (37 °C) was circulated into the scanner’s bore to keep the body temperature stable. Rats remained under anesthesia for 60 min while the anatomical MRI data were collected.

### 3.6. Immunohistochemistry

**Figure 2:**
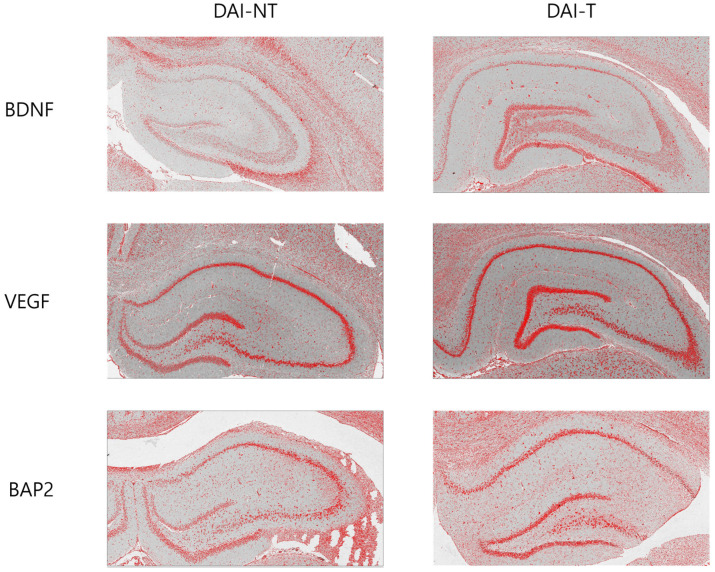
Immunohistochemical detection of the brain-derived neurotrophic factor (BDNF), vascular endothelial growth factor (VEGF), and microtubule-associated protein 2 (MAP2) expression in the hippocampus after rTMS in a DAI rat. In the immunohistochemistry for BDNF, VEGF, and BAP2 for the hippocampal area, the DAI-T group displayed the most significant immunoreactivity for BDNF, VEGF, and MAP2 compared to the DAI-NT group (×100).

All rat brains were harvested post-euthanasia for immunohistochemistry of the brain-derived neurotrophic factor (BDNF), vascular endothelial growth factor (VEGF), and microtubule-associated protein 2 (MAP2), and the tissues of the hippocampus were used. Paraffin sections were deparaffinized in xylene, hydrated, and put in phosphate-buffered saline (PBS, pH 7.6). The sections were incubated in 0.3% hydrogen peroxide for 30 min, and endogenous peroxidases were inactivated by immersion three times (five minutes each time) in 0.05 mol L-1 Tris/HCl (pH 7.4). Subsequently, they were incubated with 1% pre-immune rabbit serum to reduce non-specific staining and reacted with the primary antibody, anti-BDNF (1:500 dilution; Abcam, Waltham, MA, USA), anti-VEGF (1:500 dilution; Abcam, USA), and rabbit MAP2 (1:500, Sigma-Aldrich, St. Louis, MO, USA) in 1% bovine serum albumin (BSA), 0.1% Na-azide, and 0.3% Triton-X 100. The sections were then incubated in a moist chamber at 4.0 °C overnight. The next day, the sections were cleaned with PBS three times for 10 min each and treated with the secondary biotinylated rabbit-anti-mouse IgG antibody (1:500, Chemicon, Billerica, MA, USA). The sections were visualized with 3,3′-diaminobenzidine (DAB) substrate for two minutes and counterstained with Mayer’s hematoxylin. The ratio of immunoreactivity in the hippocampal area compared to the total area was measured with Image J (version 1.54, NIH, Bethesda, MA, USA) [23]. An X100 immunohistochemistry scan (Figure 2) was used to measure the immunoreactivity.

### 3.7. Western Blot Analysis

The western blot analysis was performed with BDNF, VEGF, and MAP2, utilizing hippocampal tissues. These tissues were homogenized in 500 μL lysis buffer, immediately chilled on ice, and preserved at −80 °C. The homogenates were centrifuged at 13,000 rpm for 10 min at 4.0 °C, and the supernatant was collected. For the detection of BDNF, VEGF, and MAP2 proteins, 20 μg of protein from each sample was resolved by 10% sodium dodecyl sulfate-polyacrylamide gel electrophoresis and subsequently transferred to polyvinylidene difluoride membranes at 300 mA for 1 h. Membrane integrity and equal protein loading were verified by Ponceau Red staining. For immunodetection, membranes were blocked in 5% non-fat milk in 20 mmol L^−1^ Tris-buffered saline containing 0.1% Tween-20 (TBS-T), 80 mmol L^−1^ NaH_2_PO_4_, 20 mmol L^−1^ Na_2_HPO_4_, and 100 mmol L^−1^ NaCl adjusted to pH 7.5 for 1 h at room temperature. After four 15 min washes in TBS-T, membranes were incubated with primary antibodies against rabbit BDNF (1:1000 dilution, Abcam, Cambridge, UK), rabbit VEGF (1:1000 dilution, Abcam, USA), and rabbit MAP2 (1:500, Sigma-Aldrich, St. Louis, MO, USA) overnight at 4.0 °C in TBS-T containing 2% non-fat milk. Following this incubation, membranes were washed three times with TBS-T for 5 min each to remove excess antibodies. Membranes were then incubated with horseradish peroxidase-conjugated goat host-rabbit IgG (1:1000 dilution, Abcam, USA) secondary antibody in a blocking solution for 30 min. After three 10 min washes in TBS-T, immunoreactive bands were detected using Enhanced Chemiluminescence Plus with Immobilon Western Chemiluminet substrate (Millipore, Billerica, MA, USA). The immunoreactive protein bands were visualized using the Enhanced Chemiluminescence Plus (ECL Plus, Amersham, UK) and Image Reader (LAS-300 Imaging System, Fuji Photo Film™, Tokyo, Japan) in grayscale at a resolution of 600 dpi and quantified using Image J (version 1.54, NIH, Bethesda, MA, USA).

### 3.8. Statistical Analyses

Statistical analyses were conducted using the SPSS software program for Windows (version 26.0, Chicago, IL, USA). Data are presented as mean ± standard deviation (SD). The repeated measure ANOVA, followed by post hoc tests, was utilized to analyze the behavioral data, and the Chi-square test was performed on the western blot data. Differences were considered statistically significant at a *p*-value of <0.05.

## 4. Results

### 4.1. Imaging Study for DAI

**Figure 3:**
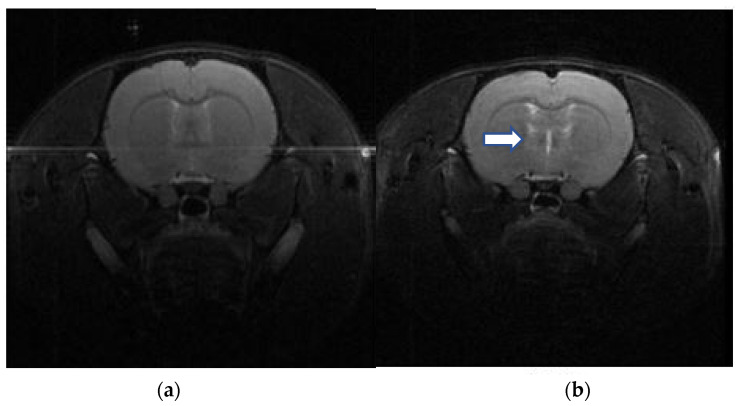
Brain MRI following modeling of a diffuse axonal injury (DAI). T2-weighted contrast MRI images display distinguishable regions. High signal intensity changes in the subcortical and deep brain tissues (white arrow) indicate the presence of DAI. (**a**) Normal rat brain MRI, (**b**) DAI rat brain MRI.

Regions identifiable by T2-weighted contrast are depicted in Figure 3. The elevated signal intensity changes in the subcortical and deep brain tissues indicate DAI following a 3.8 kg•m/s impulse over 15 s, compared to a normal rat brain MRI.

### 4.2. Behavioral Test

**Figure 4:**
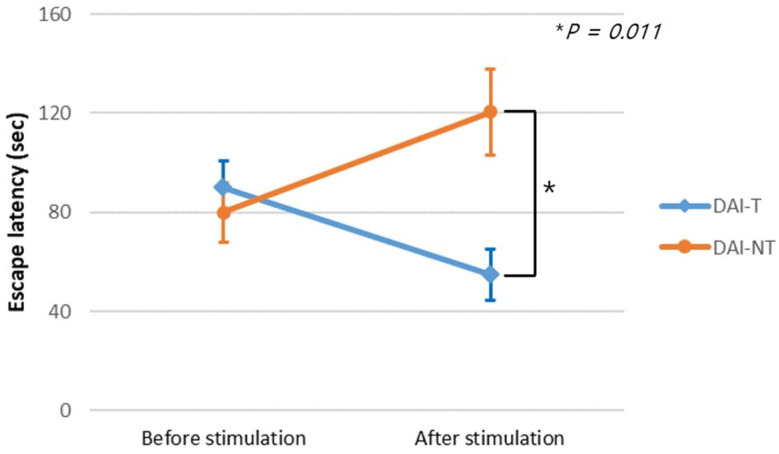
Escape latency in the Morris water maze test. Before stimulation, there was no significant difference in escape latency between the group receiving rTMS after diffuse axonal injury (DAI-T) and the no stimulation after diffuse axonal injury (DAI-NT) group; however, a significant difference emerged between the two groups after low-frequency rTMS.

The escape latency in the MWM test before the intervention was 89.72 ± 32.81 s for the DAI-T group and 79.92 ± 33.97 s for the DAI-NT group. The two groups had no significant difference (*p* = 0.556). Following the intervention, the escape latencies were 54.92 ± 30.91 s for the DAI-T group and 120.62 ± 49.03 s for the DAI-NT group. The follow-up escape latencies in the DAI-T group were significantly shorter than those in the DAI-NT group (*p* = 0.011) (Figure 4). Repeated measure ANOVA with post hoc testing showed a significant time × group interaction effect (F = 5.851, *p* = 0.009).

### 4.3. Immunohistochemistry

**Figure 5:**
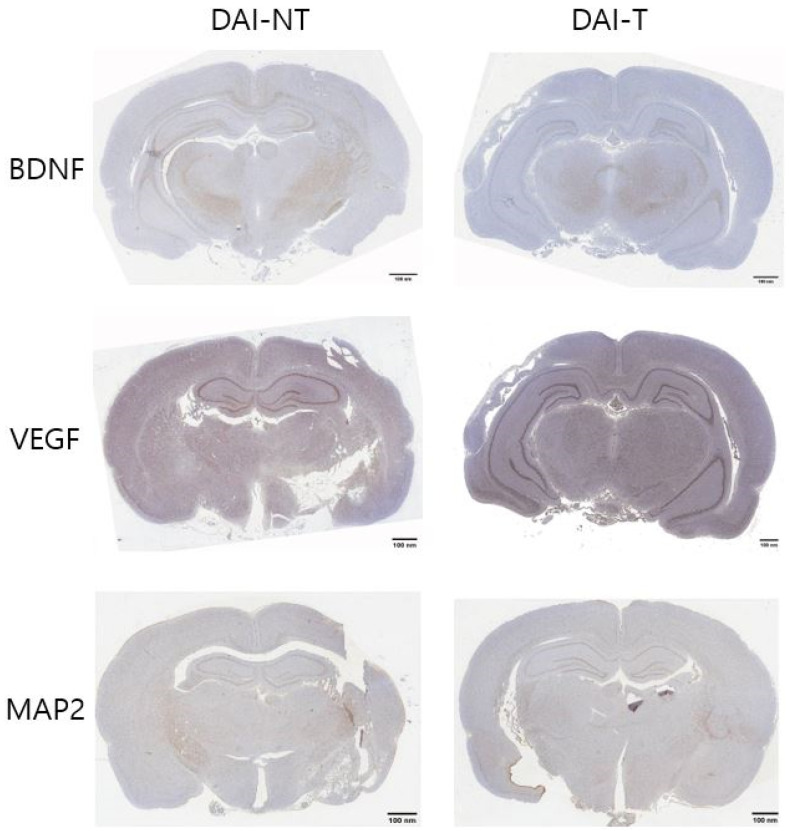
Immunohistochemical detection of the brain-derived neurotrophic factor (BDNF), vascular endothelial growth factor (VEGF), and microtubule-associated protein 2 (MAP2) expression after rTMS in a DAI rat. In the immunohistochemistry for BDNF, VEGF, and MAP2, the DAI-T group displayed the most significant immunoreactivity for BDNF, VEGF, and MAP2 compared to the DAI-NT group (×10).

Figure 2 and Figure 5 show the immunohistochemistry analysis. The ratio of BDNF immunoreactivity in the hippocampal area compared to the total area was 5.008% in the DAI-NT group and 8.778% in the DAI-T group. The ratio of VEGF immunoreactivity in the hippocampal area compared to the total area was 8.711% in the DAI-NT group and 12.105% in the DAI-T group. The ratio of MAP2 immunoreactivity in the hippocampal area compared to the total area was 10.475% in the DAI-NT group and 12.814% in the DAI-T group (Table 1).

Immunohistochemical studies showed that BDNF, VEGF, and MAP2 were more expressed in the hippocampal region adjacent to the injury in the DAI-T group than in the DAI-NT group.

### 4.4. Western Blot Analysis

Western blot analyses using antibodies against BDNF, VEGF, and MAP2 were performed on hippocampal tissues post-intervention.

**Figure 6:**
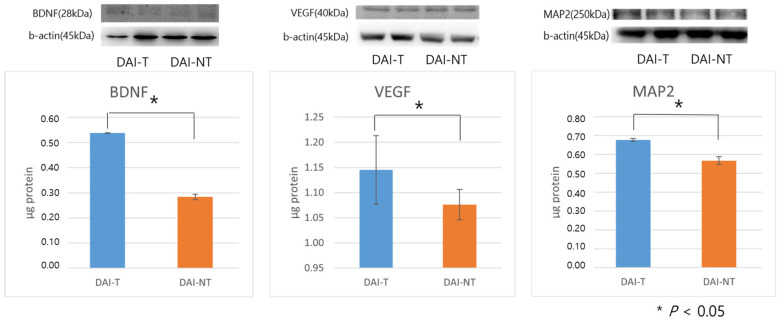
Western blot analysis of brain tissue post-intervention. The rTMS after diffuse axonal injury (DAI-T) group showed more robust expression of brain-derived neurotrophic factor (BDNF), vascular endothelial growth factor (VEGF), and microtubule-associated protein 2 (MAP2) proteins after low-frequency rTMS than the no stimulation after diffuse axonal injury (DAI-NT) group.

The BDNF protein levels were 0.284 ± 0.011 μg protein for the DAI-NT group and 0.538 ± 0.001 μg protein for the DAI-T group, indicating more robust expression in the DAI-T group (*p* = 0.000). VEGF protein levels were 1.076 ± 0.030 μg protein for the DAI-NT group and 1.145 ± 0.068 μg protein for the DAI-T group, with the DAI-T group showing more robust expression (*p* = 0.035). MAP2 protein levels were 0.567 ± 0.021 μg protein for the DAI-NT group and 0.678 ± 0.008 μg protein for the DAI-T group, again indicating more robust expression in the DAI-T group (*p* = 0.000) (Figure 6). Whole western blot bands are shown in the Supplementary File.

## 5. Discussion

DAI often lacks clear radiological changes, even in cases with prominent neurological impairment [24]. MRI can evaluate DAI in patients with non-hemorrhagic lesions who show normal findings on CT scans. In the MRI images of patients with DAI, gliosis is observed as increased T2 signals may appear in areas susceptible to shearing injury, such as the grey–white matter junction, corpus callosum, and brainstem [25]. Several rodent models of TBI share similar imaging properties with human DAI [26,27], and there have been reports that MRI is effective in evaluating the appropriateness of DAI rat models [28]. In our study, gliotic changes were observed in the grey–white matter junction and deep brain tissue on brain MRI taken after DAI modeling, confirming the appropriateness of the modeling.

This study reported the effects of low-frequency rTMS applied to the frontal cortex using a figure-eight coil at 1 Hz in DAI rats. rTMS has been shown to effectively treat cognitive disorders [29]. Additionally, previous research indicates that high-frequency rTMS may benefit cognition in patients with mild cognitive impairment or Alzheimer’s disease [30]. However, the impact of low-frequency rTMS on cognitive function in DAI remains controversial, with results showing inconsistency [31].

rTMS can produce various long-term effects based on intensity, frequency, and stimulation patterns. High-frequency stimulation (>3 Hz) typically leads to facilitation, a phenomenon similar to long-term potentiation (LTP). In contrast, low-frequency rTMS (≤1 Hz) results in a reduction in synaptic efficiency, akin to long-term depression (LTD) [32]. This study observed a significant difference in escape latency between two TBI rat groups post-intervention. DAI involves microscopic and gross damage to axons resulting from acceleration and deceleration motions that generate shearing forces on the brain’s white matter tracts [33]. This process is associated with an imbalance between glutamate and GABA. In the acute phase of TBI, excitotoxic injury extends to adjacent tissues, and excessive glutamate release increases intracellular calcium levels, furthering neuronal damage [34,35]. Therefore, considering the neuronal harm caused by cortical hyperexcitability in DAI, it is proposed that cortical inhibition via low-frequency rTMS may contribute to the observed difference in escape latency. Consequently, neuromodulation through low-frequency rTMS is suggested to have a protective effect on the inflammatory response following acute brain injury.

This study revealed elevated expression levels of BDNF, VEGF, and MAP2 in the hippocampus of the DAI-T group. The mechanism behind DAI remains elusive; however, axoplasmic calcium overload is believed to play a critical role in axonal injury. Furthermore, disturbances in axonal calcium homeostasis are pivotal in axonal damage during the acute phase of DAI [36]. Therefore, managing the calcium signaling pathway is vital for patients with DAI.

BDNF, acting as a neuroprotective agent, promotes neuroprotection by regulating Bcl-2 family proteins [37]. The Bcl-2 family proteins, known for their significant neuroprotective role, can influence the intracellular Ca^2+^ transport systems [38]. A previous study indicated a notable increase in Bcl-2 expression following low-frequency rTMS in dementia rats [39]. It is hypothesized that low-frequency rTMS may enhance proteins involved in calcium signaling pathways, such as Bcl-2, thereby preventing axonal injury resulting from excitotoxicity.

MAP2, a prominent cytoskeletal component, is crucial for microtubule stability and neural plasticity as it supports microtubule stabilization [40]. A key mechanism of DAI involves cytoskeletal alterations in axons due to acceleration and deceleration forces [41]. A recent report showed the potential mechanisms by which MAP2 can be regulated via post-translational modification to enhance the neuroprotective process [42]. Intermittent theta-burst rTMS as a high-frequency protocol may inhibit the ischemic and reperfusion injury related to the expression of MAP2 protein [43]. Also, low-frequency rTMS could induce changes in MAP-2 expressions in brain structures to affect synaptic plasticity [44]. Both low- and high-frequency rTMS could affect neuroplasticity, but low-frequency rTMS may be related to the neuroprotective mechanism after DAI, according to this study.

VEGF, recognized for its angiogenic and vascular permeability-enhancing properties, also offers neuroprotective benefits through apoptosis inhibition and neurogenesis stimulation, preventing neurons from dying under critical conditions [45]. Also, VEGF expression increased in a rat model of vascular dementia after 1 Hz rTMS [39]. This study revealed that low-frequency rTMS may have neuroprotective effects on DAI rat models like the vascular dementia rat models.

This study presents several limitations. First, the experiment utilized a small sample size of rats. Future studies may need a larger sample size to enhance statistical power and robustness. Second, the evaluation of DAI was exclusively conducted through brain MRI without assessing its severity. Given the findings indicating that cognitive function in patients with depressive disorders can serve as an indicator to predict the response to rTMS [46], further research is needed to explore the therapeutic effects of rTMS in DAI according to its severity. Third, the control group was in the same circumstances as the DAI-T group during the experiment. However, there was no sham rTMS applied. Last, our study only examined the short-term effects of rTMS on cognition. Considering the potential neuroprotection effect of low-frequency rTMS in acute DAI, as suggested by our study results, it would be necessary to investigate its long-term effects as well.

Despite these limitations, the current study identified significant alterations in escape latency following low-frequency rTMS, thus highlighting the efficacy of the molecular–biological approach. In other words, our study demonstrated that BDNF, MAP2, and VEGF increased following low-frequency rTMS. BDNF supports learning and memory through synergistic interactions between neuronal activity and synaptic plasticity [47], MAP2 influences neuronal plasticity through its involvement in the initial differentiation of neurons [48], and VEGF is recognized for its neuroprotective effects against excitotoxicity associated with neurodegeneration [49]. BDNF, MAP2, and VEGF each play a unique role, but they also interact to protect neurons and maintain cognitive function. Elevated expression levels of BDNF, VEGF, and MAP2 after rTMS may align with the neuroprotective impact of low-frequency rTMS and suggest a molecular biomechanism for the protective effect against the inflammatory process following acute brain injury. Therefore, there is a need for further research into strategies for enhancing cognitive function in patients with DAI through the appropriate regulation and interaction of these molecules.

Consequently, further studies are warranted to elucidate the mechanism by investigating the effects of various non-invasive brain stimulation techniques compared to rTMS following DAI. Additionally, it is essential to understand potential challenges or safety issues that may arise when applying these techniques to humans.

## 6. Conclusions

The application of rTMS enhanced cognitive functions in a rat model of DAI, demonstrating increased expression of BDNF, VEGF, and MAP2. These findings potentially support the use of rTMS to treat patients with cognitive deficits attributable to DAI.

## Figures and Tables

**Table 1:** The ratio of immunoreactivity after intervention.

Immunohistochemistry	Group	Area	Mean	% Area
BDNF	DAI-NT	1,654,224	202.597	5.008
	DAI-T	1,654,224	192.308	8.778
VEGF	DAI-NT	1,654,224	168.547	8.711
	DAI-T	1,654,224	158.346	12.105
MAP2	DAI-NT	1,654,224	204.679	10.475
	DAI-T	1,654,224	202.148	12.814

BDNF, brain-derived neurotrophic factor; VEGF, vascular endothelial growth factor; MAP2, microtubule-associated protein 2; DAI-NT, no stimulation after diffuse axonal injury; DAI-T, rTMS after diffuse axonal injury.

## Data Availability

The data presented in this study are available on request from the corresponding author.

## Supplementary Materials

The following supporting information can be downloaded at: https://www.mdpi.com/article/10.3390/neurolint16040052/s1, Figure S1: Whole western blot bands.
