# Supplementary figures and images for: The Effect of Repetitive Transcranial Magnetic Stimulation on Cognition in Diffuse Axonal Injury in a Rat Model
Source: Neurol Int. 2024 Jun 25;16(4):689–700. doi: 10.3390/neurolint16040052 (PMC11270180; doi:10.3390/neurolint16040052)

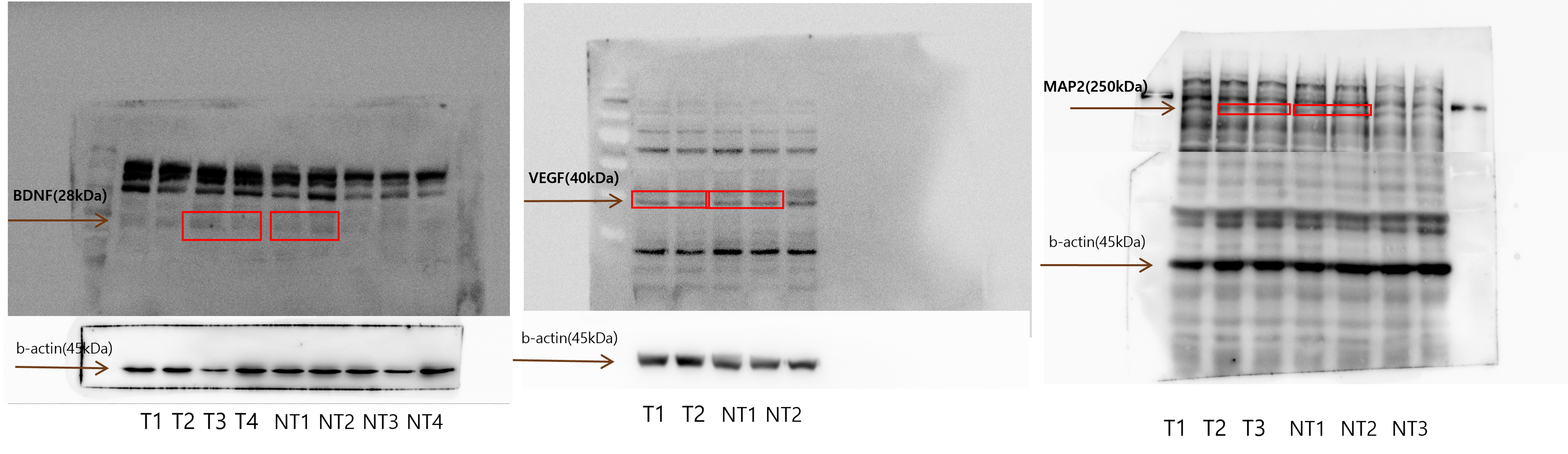

Supplement: Supplementary file 1 [file neurolint-16-00052-s001.zip › neurolint-2986530-supplementary.tif]
